# Metastasis-associated protein 2 regulates human hepatocellular carcinoma metastasis progression through modulating p38MAPK/MMP2 pathways

**DOI:** 10.7150/jca.35626

**Published:** 2019-10-22

**Authors:** Wen-Hung Hsu, Hui-Ling Chiou, Chia-Liang Lin, Shao-Hsuan Kao, Hsiang-Lin Lee, Chung-Jung Liu, Yi-Hsien Hsieh

**Affiliations:** 1 Division of Gastroenterology, Department of Internal Medicine, Kaohsiung Medical University Hospital, Kaohsiung, Taiwan; 2 School of Medical Laboratory and Biotechnology, Chung Shan Medical University, Taichung, Taiwan; 3 Institute of Biochemistry, Microbiology, and Immunology, Chung Shan Medical University, Taichung, Taiwan; 4 Institute of Medicine, Chung Shan Medical University, Taichung, Taiwan.; 5 Department of Surgery, Chung Shan Medical University Hospital, Taichung, Taiwan; 6 Center for Stem Cell Research, Kaohsiung Medical University, Kaohsiung, Taiwan; 7 Department of Biochemistry, School of Medicine, Chung Shan Medical University, Taichung, Taiwan; 8 Clinical laboratory, Chung Shan Medical University Hospital, Taichung, Taiwan

**Keywords:** Hepatocellular carcinoma cell, MTA2, Migration, Invasion, p38MAPK, MMP-2

## Abstract

Studies have shown the overexpression of metastasis-associated protein 2 (MTA2) to be associated with hepatocellular carcinoma (HCC) progression. However, the molecular mechanism of MTA2 expression in HCC is unclear. In our study, we found a higher level of MTA2 in HCC tissues than in normal tissues and a significant correlation between tumor grade and overall survival of HCC patients. We also found that MTA2 inhibition reduced the migration and invasion capabilities of HCC cells, independent of cell proliferation. Mechanistic studies have suggested that MTA2 protein and mRNA are more highly expressed in SK-Hep-1 and Huh-7 cells compared with other HCC cells. MTA2 silencing drastically reduced migration and invasion capability and also inhibited matrix metalloproteinase 2 (MMP2) at the transcriptional and translation levels in both cells. In addition, treatment with the MMP2 antibody markedly impaired MTA2-knockdown-mediated inhibition of migration and invasion in SK-Hep-1 cells. Furthermore, MTA2 knockdown reduced the phosphorylation of the p38MAPK protein, whereas the inhibition of p38MAPK (SB203580 or si-p38) confirmed that blocking the p38MAPK pathway mediated MTA2-knockdown-inhibited migration and invasion in SK-Hep-1 cells. We demonstrated the molecular mechanism by which MTA2 inhibits human HCC cell metastasis through the p38MAPK/MMP2 pathways, which might be helpful in determining the diagnostic value of this protein in patients with HCC

## Introduction

Hepatocellular carcinoma (HCC), one of the most prevalent cancers, is one cause of the high cancer-related mortality in developed countries 1. HCC accounts for more than 700,000 new cases of cancer each year. Despite advanced treatments, including chemotherapy, radiotherapy, and surgical intervention, HCC causes more than 600,000 deaths worldwide every year 1, 2. Chemotherapeutic resistance, invasion, and metastasis of cancer cells correlate with aggressive progression and lead to a lack of effective therapy and low overall survival (OS) in patients with HCC 2. Therefore, new potential approaches for treating HCC must be explored through careful study of relevant molecular features to identify therapeutic targets for HCC.

Metastasis of cancer cells through migration, invasion, and the related degradation of the extracellular matrix (ECM) exacerbate tumor development. Malignant cells undergo several changes for distant metastasis to occur. First, cancer cells obtain the ability to migrate and invade the surrounding tissues. Second, they destroy intercellular interactions (tight junctions and gap junctions). Third, the structure of the ECM is further lysed or destroyed. Finally, the adhesive ability of cells to the ECM is upregulated 3. ECM degradation by extracellular proteinases contributes to the progression of tumor cell invasion and metastasis 4. Among proteolytic proteinase systems, matrix metalloproteinases (MMPs) play a critical role in degrading the ECM. ECM degradation by MMP2 or MMP9 allows metastatic cells to invade and migrate into the target organ, contributing to tumor metastasis 5, 6.

Metastasis-associated protein 2 (MTA2), a 668-amino-acid protein, appears to augment the potential of cancer cells by regulating cytoskeletal organization 7. MTA2 regulates the activation of cytoskeletal pathways at the transcriptional modification phase 8. MTA2-overexpressing cells exhibit upregulation in cytoskeletal change, focal adhesion, and Rho activation 9. A high expression of MTA2 also contributes to lamellipodia formation in breast cancer cells 10. MTA2 overexpression enhances metastatic behavior *in vitro* and *in vivo* and is associated with poor outcomes in estrogen-receptor-negative breast cancer 11. MTA2 also regulates the activity of Twist, which is an essential factor for epithelial-mesenchymal transition 12. MTA2 knockdown suppresses the proliferation and invasion of human glioma cells *in vitro* and *in vivo* 13.

In the present study, the expression of MTA2 in human HCC tissues was measured, and the relationship between the MTA2 expression and OS of patients with HCC was investigated. Additionally, MTA2's role in cell cycle regulation as well as cell growth, migration, and invasion were examined, and the precise molecular mechanism behind this regulation in human HCC cells was identified. The results demonstrated that MTA2 knockdown considerably inhibited invasive motility by suppressing MMP2 expression and downregulating the p38 signaling pathway. Taken together, MTA2 could be helpful if used as a diagnostic factor and could serve as a novel therapeutic target for HCC.

## Materials and Methods

### Human hepatocellular carcinoma tissue array and immunohistochemistry (IHC) Staining

A human HCC tissue array was purchased from US BioMax (LV1002). Immunohistochemistry (IHC) staining was performed on sections of paraffin-embedded tissues. Formalin-fixed prostate tissues were embedded in paraffin, and 3-µm sections were prepared from tissue samples. The sections were further processed by heating at 70 °C in an oven for 30 min and dewaxed with xylene and alcohol for 10 min. Endogenous peroxidase was blocked for 20 min in 0.3% H_2_O_2_. The tissues were treated with citrate buffer for 10 min at 95 °C. Anti-MTA2 antibody (sc-55566; 1:100; Santa Cruz, CA, USA) was the primary antibody used for IHC staining. DAKO EnVision containing horseradish peroxidase conjugated with antigoat antibodies was incubated for 30 min, after which the sections were visualized using a diaminobenzidine solution. Finally, the quantification of protein expression in the IHC staining was evaluated according to the immunoreactivity score.

### The Cancer Genome Atlas Database

Using The Cancer Genome Atlas (TCGA) database, the expression of MTA2 in patient HCC tissues was analyzed. HCC patients were divided into low- and high-expression groups, and the values of MTA2 as they relate to differential OS analysis and tumor grade were examined.

### Cell Culture

Five HCC cell lines were used in subsequent experimental investigations. SK-Hep-1 cells were purchased from the American Type Culture Collection (Rockville, MD, USA). Huh-7 cells were a gift from Dr. Hui-Ling Chiou (Chung Shan Medical University, Taichung, Taiwan). HepG2, PLC/PRF/5, and HA22T/VGH cells were purchased from the Bioresource Collection and Research Center and the Food Industry Research and Development Institute (Hsinchu, Taiwan). The SK-Hep-1, HuH-7, and HA22T/VGH cells were cultured in Dulbecco's modified Eagle's medium (DMEM), whereas the PLC/PRF/5 and HepG2 cells were cultured in minimum essential medium. The PLC/PRF/5 and HepG2 cells were supplemented with 10% fetal bovine serum (Hyclone Laboratories, Logan, UT, USA), 100 μg/mL NEAA, and 100 mg/mL penicillin-streptomycin (Sigma Chemicals, St. Louis, MO, USA). These cell lines were maintained in a humidified atmosphere containing 5% CO2 at 37 °C.

### shRNA-MTA2 lentivirus infection

MTA2 shRNA lentiviral constructs were purchased from the National RNA Interference Core Facility (Institute of Molecular Biology, Academia Sinica, Taipei, Taiwan). The transfection assay was performed as Institutional Biosafety Committee of Chung Shan University. HEK293T cells were used for target lentivirus production. Both SK-Hep-1 cells and Huh-7 cells with stable MTA2 knockdowns were generated by lentiviral infection and then followed by selection with 2 μg/mL puromycin for 2 weeks. Inhibition efficiency of the shRNA-MTA2 construct was confirmed through immunoblotting and reverse-transcription quantitative polymerase chain reaction (RT-qPCR) analysis.

### Cell Viability Assay

Cell viability was analyzed using an MTT assay. The absorbance of blue formazan crystals was measured at 570 nm using an enzyme-linked immunosorbent assay plate reader. The quantity of the formazan product was directly proportional to the number of viable cells in the culture medium. The viability of the HCC cells was determined according to the absorbance, which was corrected in accordance with a background reading.

### Flow Cytometric Analysis

Cells were centrifuged at 800 rpm at 4 °C for 5 min, washed with ice-cold phosphate-buffered saline (PBS), and stained with a propidium iodide (PI) buffer (4.0 g/mL PI, 1% Triton X-100, 0.5 mg/mL RNase A in PBS). The cells then were analyzed and measured using a Muse Cell Analyzer (Merck, Millipore).

### *In vitro* Migration and Invasion Assay

Cell migration and invasion assays were performed using 24-well modified Boyden chambers containing membrane filter inserts with 8-μm pores (Corning Incorporated Life Sciences, Tewksbury, MA, USA). Membrane filter inserts were precoated with Matrigel for the invasion assay, and the lower compartment was filled with DMEM containing 20% fetal bovine serum. Huh-7 and SK-Hep-1 cells were placed in the upper part of a Boyden chamber containing serum-free medium and were incubated for 16-24 h. Migratory and invasive phenotypes were determined by counting the cells that had migrated to the lower side of the filter through microscopy at 100-fold magnification. The third fields were counted for each filter and measured in triplicate.

### Immunoblotting

Cells were washed with cold PBS and resuspended in lysis buffer with a cocktail (Roche Molecular Biochemicals). After 20 min of incubation, the supernatant was collected through centrifugation at 12,000 g for 15 min at 4 °C, and the protein concentration was determined using the Bradford method. Equal amounts of protein were loaded and analyzed using immunoblotting. Briefly, proteins were separated by 10% sodium dodecyl sulfate polyacrylamide gel (SDS-PAGE) electrophoresis and transferred onto a polyvinylidene fluoride membrane (PVDF; Life Technologies, Carlsbad, CA, USA). The membranes were blocked with a nonfat dry milk buffer (5% nonfat dry milk) for 2 h at room temperature. Then, the membranes were incubated with primary antibodies, including anti-MTA2 (1:1000; sc-55566), anti-MMP2 (1:1000; sc-53630), anti-MMP9 (1:500; sc-21733), anti-pERK (1:1000; sc-136521), anti-ERK (1:1000; sc-514302), anti-pp38 (1:1000; sc-166182), anti-p38 (1:1000; sc-7972) and β-actin (1:2000; sc-69879) in the aforementioned solution on an orbital shaker at 4 °C overnight. Following primary antibody incubations, the membranes were incubated with horseradish-peroxidase-linked secondary antibodies (anti-rabbit, -mouse, or -goat IgG). Antibody-bound protein bands were detected using an enhanced chemiluminescence reagent (Millipore, Billerica, MA, USA) and were photographed with an ImageQuant LAS 4000 Mini imaging system.

### Reverse transcription and real-time PCR assay

Total RNA was isolated from the cultured cells. The cells were homogenized in Isol-RNA-Lysis Reagent (Gaithersburg, MD, USA), and a reverse-transcription assay was performed using GoScript Reverse Transcriptase (Madison, WI, USA). The qPCR result was analyzed using a StepOne Real-Time PCR System (Applied Biosystems, Foster City, California, USA). The primers were as follows: the human MTA2 forward primer was 5'-TGAGATGGAGGAATGGTCAGCC-3', and the reverse primer was 5'-CTGGACTATGCTGGCAAGTGAC-3'; the human MMP2 forward primer was 5'-TGGCAAGTACGGCTTCTGTC-3', and the reverse primer 5'-TTCTTGTCGCGGTCGTAGTC-3'; human glyceraldehyde 3-phosphate dehydrogenase (GAPDH) forward primer was 5'-CATCATCCCTGCCTC TACTG-3', and the reverse primer was 5'-GCCTGCTTCACCACCTTC-3' (Mission Biotech, Taipei, Taiwan). Relative gene expression was normalized with endogenous GAPDH and analyzed using the 2-^ΔΔCt^ method.

### siRNA-p38 transfection

The siRNA specifically targeting p38 (si-p38) and a scrambled control siRNA were commercially constructed by and obtained from AllBio Science, Inc (Taipei, Taiwan). The SK-Hep-1 and Huh-7 cells were plated and cultured in a medium in a 6-cm culture dish before siRNA transfection using Lipofectamine RNAiMAX Transfection Reagent (Thermo Fisher Scientific, Waltham, MA, USA) was performed according to the manufacturer's protocol. The si-p38: 5'-GCCACCAAGAUGCUGACAUTT-3' was the major target sequence for p38MAPK.

### Promoter luciferase Reporter Gene Assay

Human stable MTA2 knockdown SK-Hep-1 and Huh-7 cells were transfected with human MMP2-promoter-luciferase plasmid and beta-gal plasmid. The beta-gal plasmid acted as a control for evaluating transfection efficiency. At 36 h after transfection, the MMP2-promoter-luciferase activity assay and β-gal enzyme assay were performed according to the instructions of the luciferase assay kit (Promega, Madison, WI, USA).

### Statistical Analysis

Statistical analyses were performed using SPSS 20 statistical software, and the significance of differences between each group was analyzed using Student's t test. The survival curves of HCC patients were measured using the Kaplan-Meier method and log-rank test. All results are presented as mean ± standard deviation, and significance was defined as P < 0.05 or P < 0.01.

## Results

### Expression of MTA2 is Significantly Correlated with Survival in HCC Patients

**Figure 1:**
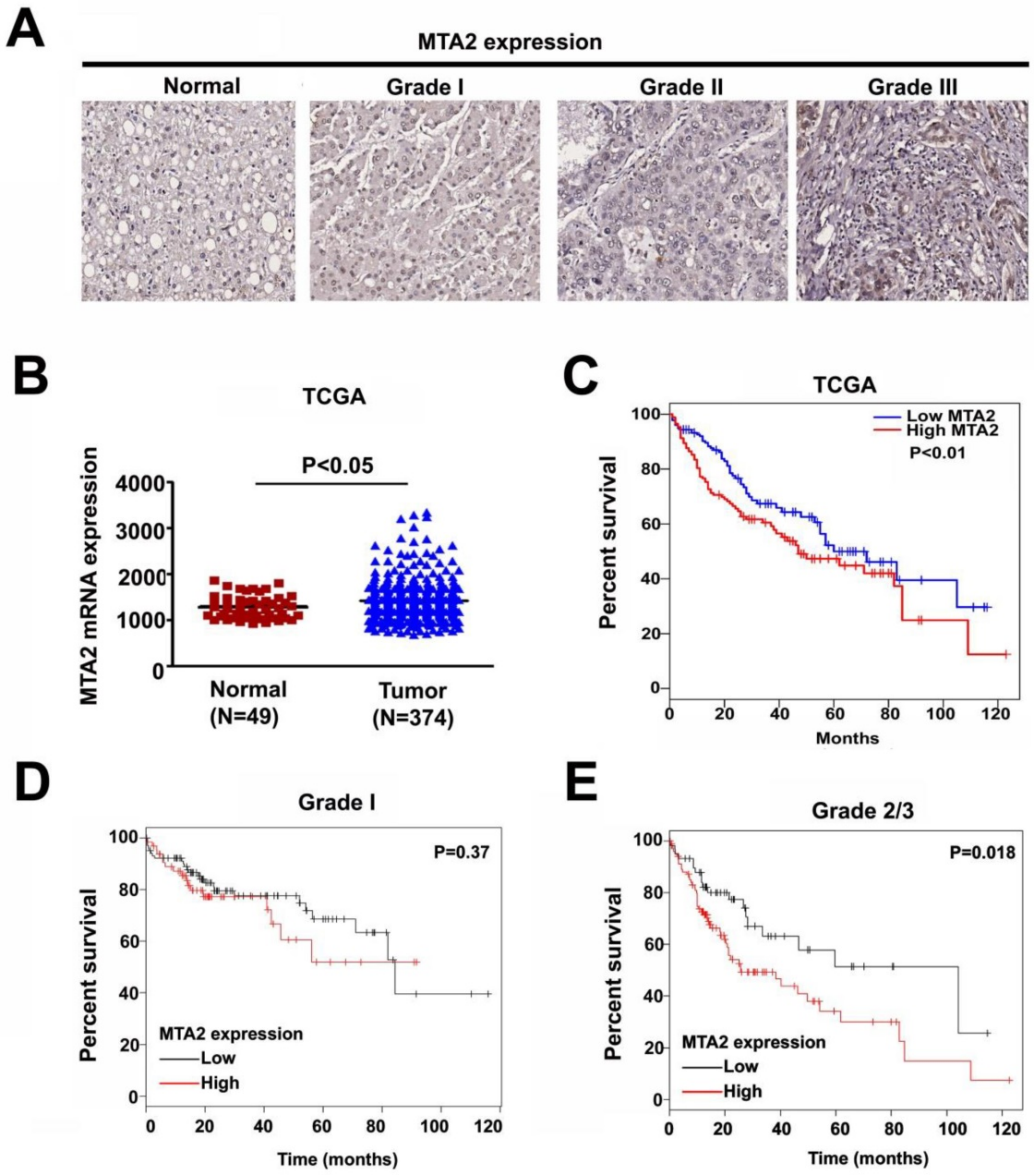
Upregulation of MTA2 in HCC tissues associated with poor prognosis. (A) IHC analysis of MTA2 expression in normal liver tissues and tissues at different stages of HCC (stages 1-3). (B) Relative mRNA expression of MTA2 in normal liver tissues (N=49) and HCC tissues (N=374) from TCGA database analysis. (C) Kaplan-Meier survival curves regarding the low and high expression of MTA2 in HCC tissues from TCGA database analysis. (D.E) OS for HCC patients with a high or low expression of MTA2, as correlated with low- (grade-1) or high-grade (grade-2/3) HCC tissues according to TCGA database. *P < 0.05 and **P < 0.01 indicate a significant difference.

To clarify the role of MTA2 in HCC, a human HCC tissue array was analyzed to detect MTA2 expression through IHC assay. As shown in Figure 1A, higher MTA2 protein expression was found in HCC tissues than in normal liver tissues (P < 0.01), and similar results are found in TCGA database (P < 0.05; Figure 1B). A high expression of MTA2 was found to be significantly correlated with only the tumor grade of HCC patients (P < 0.01; Table 1). Next, we analyzed the correlations between MTA2 and clinicopathological features and OS in HCC patients. As shown in Figure 1C, the OS of patients was higher in HCC tissues with low MTA2 compared with high expression of MTA2 (P < 0.01). A Kaplan-Meier survival analysis showed that low expression of MTA2 was significantly correlated with OS in grade-2/3 HCC tissue compared with grade-1 HCC tissues (Figures 1D and 1E). The results suggest that MTA2 could be a predictive factor for HCC patients.

### MTA2 expression in human HCC cell lines

**Figure 2:**
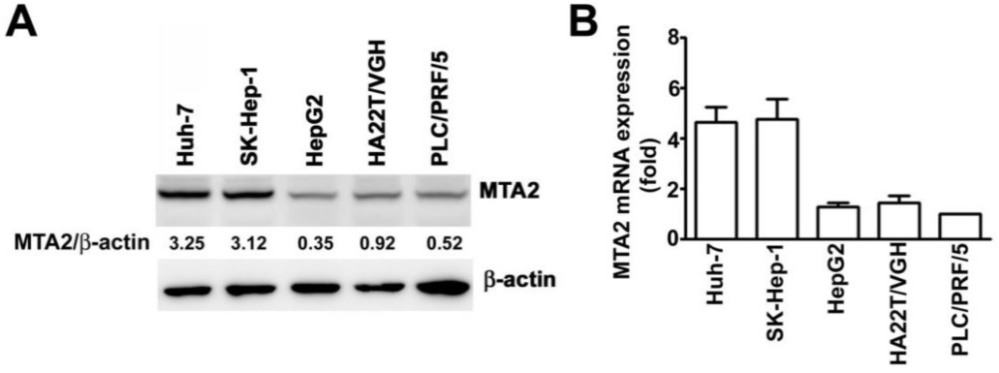
MTA2 expression in HCC cell lines. MTA2 expression was considerably higher in SK-Hep-1 and Huh-7 cell lines compared with other HCC cells (PLC/PRF/5, HepG2m, and HA22T/VGH), as measured through (A) Western blotting and (B) RT-qPCR assay. β-actin and GAPDH are presented as protein and mRNA loading controls, respectively.

To examine the expression of MTA2 in HCC cells, SK-Hep-1 and Huh-7 cells (cells with high MTA2 expression) as well as PLC/PRF/5, HepG2, and HA22T/VGH cells (cells with low MTA2 expression) were further investigated using Western blotting and an RT-qPCR assay. High protein and mRNA levels of MTA2 in the Huh-7 and SK-Hep-1 cells were found compared with the PLC/PRF/5, HepG2, and HA22T/VGH cells (Figures 2A and 2B). Therefore, Huh-7 and SK-Hep-1 cell lines were used in the subsequent investigations.

### Effect of MTA2 knockdown on cell growth in human HCC cells

**Figure 3:**
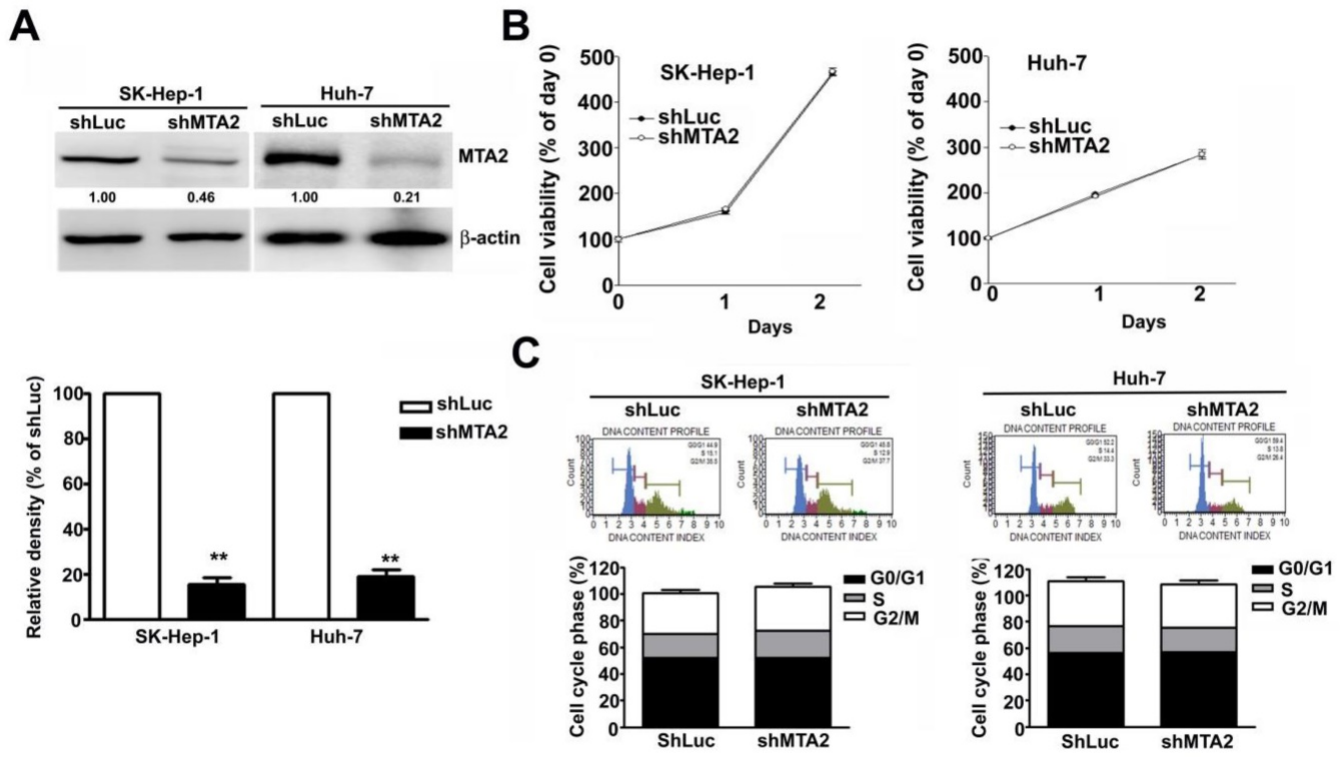
Effect of MTA2 knockdown on cell viability in human HCC cells. (A) Western blot shows MTA2 protein level in MTA2 knockdown SK-Hep-1 and Huh-7 cells. (B) Cell viability of the MTA2 knockdown cells was measured using MTT assay. (C) PI-stained cells show the analysis of cell cycle distribution rates using a flow cytometry assay. **P < 0.01 indicates a significant difference, compared with shLuc cells.

The aberrant expression of MTA2 in HCC cells indicates that MTA2 is involved in HCC progression. The effect of MTA2 knockdown on cell growth in human HCC cell lines SK-Hep-1 and Huh-7 was observed. The reduced MTA2 expression in MTA2-knockdown SK-Hep-1 cells and Huh-7 cells, compared with shLuc cells, was confirmed through immunoblotting (Figure 3A). The growth of these cells was then measured using MTT assay. As shown in Figure 3B, MTA2 knockdown showed no inhibitory effect on the growth of HCC cells. A flow cytometry assay was then performed to confirm the MTA2 knockdown did not affect the cell cycle (Figure 3C). The results showed that MTA2 inhibition had no significant effect on HCC cell growth.

### MTA2 Knockdown Inhibits Cell Migration and Invasion in Human HCC Cells

**Figure 4:**
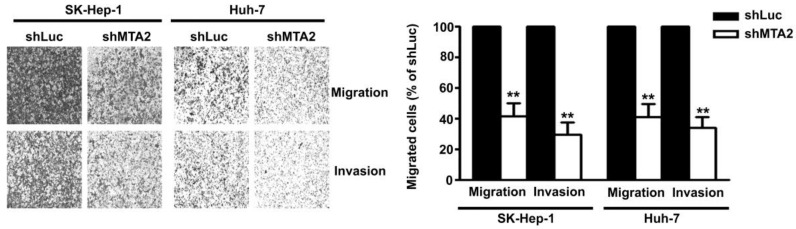
Effect of MTA2 knockdown on cell migration and invasion in HCC cell lines. Representative micrographs (left panel) and quantification (right panel) of MTA2 knockdown SK-Hep-1 and Huh-7 cells by *in vitro* migration and Matrigel-coated invasion assay. **P < 0.01 indicates a significant difference, compared with shLuc cells.

To identify the effect of MTA2 knockdown on cellular migration and invasion ability in human HCC cells, an *in vitro* migration and invasion assay was performed in Huh-7 and SK-Hep-1 cells. As shown in Figure 4, MTA2 knockdown drastically reduced the migratory and invasive capacity of Huh-7 and SK-Hep-1 cells. These results indicate that MTA2 plays a vital role in the tumor metastasis of HCC and that MTA2 knockdown suppresses the migration and invasion of human HCC cells.

### MTA2 Knockdown Inhibits Transcription and Translation Activities of MMP2 in HCC Cells

**Figure 5:**
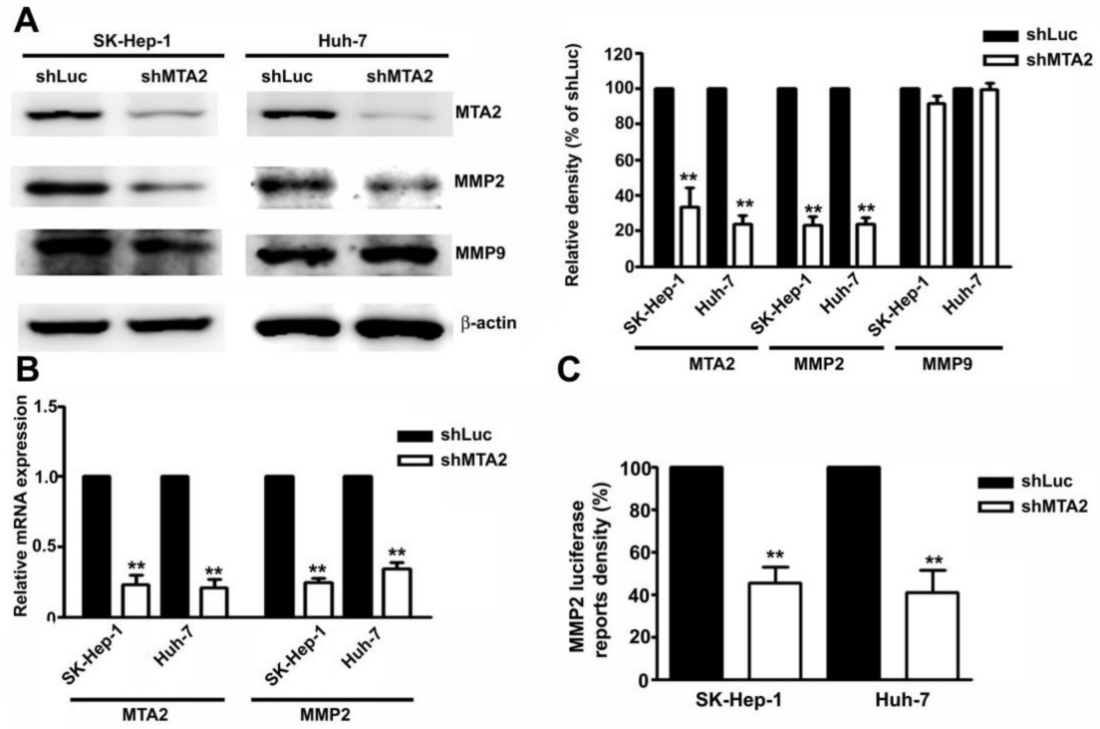
Knockdown of MTA2 inhibits the expression of MMP2 in HCC cells.

The role of MMP2 and MMP9 in MTA2-knockdown-inhibited migration and invasion in human SK-Hep-1 and Huh-7 HCC cells was identified. Knockdown of MTA2 considerably downregulated the protein and mRNA levels of MMP2 but did not affect MMP9 expression (Figures 5A and 5B). To investigate the effect of MTA2 knockdown on the regulation of MMP2 transcriptional activity, transient transfection was performed using human MMP2-luciferase-promoter constructs in human SK-Hep-1 cells and Huh-7 cells with MTA2 knockdown. It was determined that MTA2 knockdown considerably reduced MMP2 promoter activity compared with that of shLuc cells (Figure 5C). These findings suggested that MMP2 is involved in MTA2-regulated cell migration and invasion in human HCC cells.

Protein expression of MTA2, MMP2, and MMP9 was measured using Western blotting. (B) RT-qPCR assay was used to determine the mRNA expression of MTA2 and MMP2. (C) The promoter activity of MMP2 was measured using luciferase reporter assay. **P < 0.01 indicates a significant difference compared with shLuc cells.

**Figure 6:**
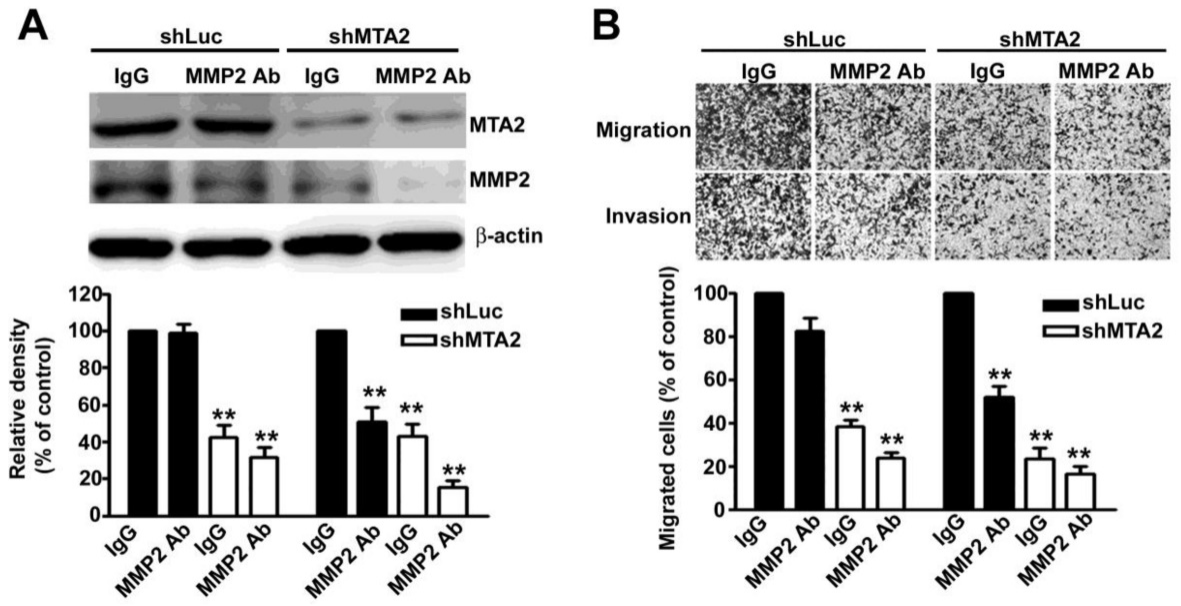
MMP2 is involved in HCC cell migration and invasion. (A) Treatment with and without MMP2 antibody (MMP2 Ab) in shLuc- or shMTA2-HCC cells. Western blotting assay measured MTA2 and MMP2 expression. (B) Representative micrographs (upper panel) and quantification (lower panel) of cell migration and invasion. **P < 0.01 indicates a significant difference compared with shLuc cells.

To thoroughly verify the role of MMP2 in MTA2 knockdown effects, we found that treatment with an MMP2 antibody (MMP2-Ab) in knockdown MTA2 cells considerably reduced the expression of MMP2 (Figure 6A) as well as inhibited cell migration and invasion (Figure 6B) compared with rabbit IgG antibody (control). Therefore, these results suggest that MMP2 is essential for the metastasis effect of MTA2 on HCC progression.

### MTA2 is involved in the p38MAPK Pathway in HCC Cells

**Figure 7:**
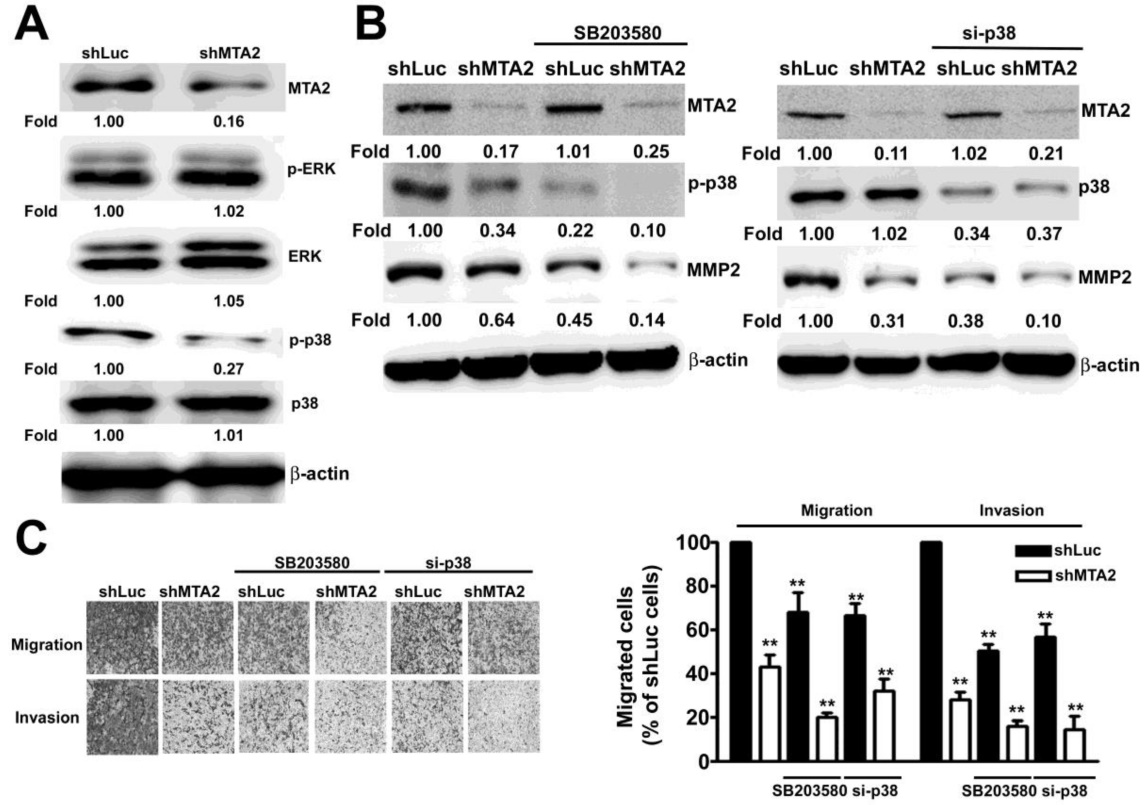
p38MAPK is involved in MTA2 regulation of cell migration and invasion. (A) The expression of MTA2, p-ERK, EKR1/2, p-p38, and p38 in shLuc or shMTA2 cells was evaluated using Western blotting. (B) shLuc or shMTA2 cells were pretreated with the SB20358 or p38MAPK siRNA (si-p38); this was followed by western blotting, and (C) *in vitro* migration and invasion assays. **P < 0.01 indicates a significant difference compared with shLuc cells.

To identify the ERK1/2 and p38MAPK signaling pathways that are involved in MTA2-regulated cell migration and invasion in human HCC cells, the phosphorylation of the ERK1/2, JNK1/2, and p38MAPK signaling pathways was measured using Western blotting. As shown in Figure 7A, MTA2 knockdown considerably suppressed the phosphorylation of the p38MAPK signaling pathway in Huh-7 and SK-Hep-1 cells. To determine the functional relevance of the p38MAPK pathway in MTA2-knockdown SK-Hep-1 cells, treatment with 20 μM SB203580 (specific p38MAPK inhibitor) or 100 nM siRNA-p38 (si-p38) was used in further study. Pretreated with SB203580 or si-p38 in MTA2 knockdown cells were significantly inhibited the expression of MMP2 as compared to that shLuc cells or treated with SB203580 or si-p38 alone, respectively (Figure 7B). As shown in Figure 7C, intercepting p38MAPK signaling significantly reduced the migration and invasion inhibition caused by MTA2 knockdown in SK-Hep-1 cells. These results demonstrated that MTA2 knockdown inhibited the metastasis of HCC cells through the inactivation of the p38MAPK pathways *in vitro*.

### Correlations of Levels of MTA2 with MMP2 and p38MAPK Expression in HCC Patients

**Figure 8:**
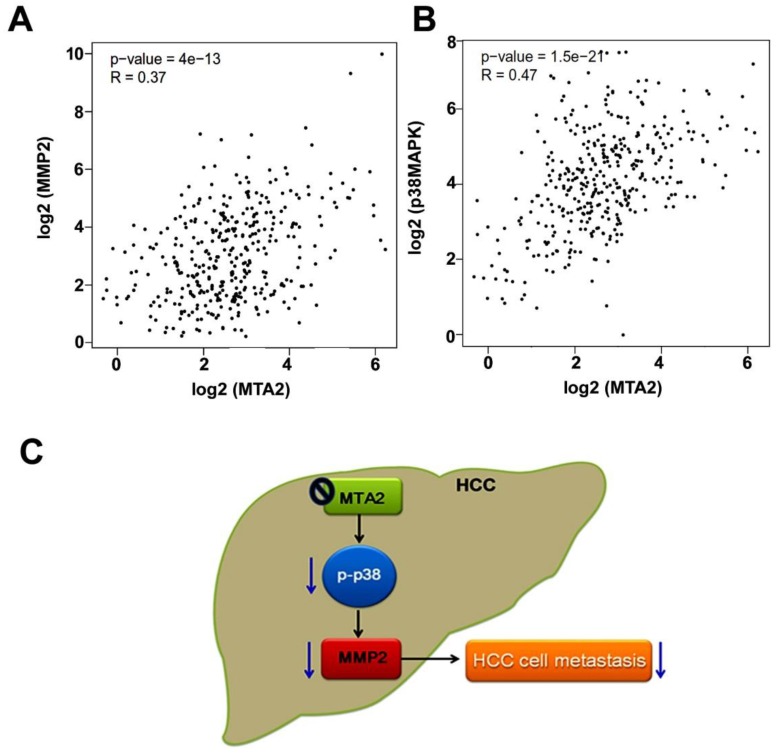
MTA2 correlation with MMP2 and p38MAPK in HCC tissues. (A, B) GEPIA database revealed that MMP2 and p38MAPK levels are positively correlated with MTA2 expression in HCC tissues. P < 0.01 indicates a significant difference. (C) Graphical summary of MTA2 inhibits human HCC cell metastasis through the p38MAPK/MMP2 signaling pathways.

Whether MTA2 levels are associated with MMP2 or p38MAPK expression in TCGA database was evaluated using GEPIA (Gene Expression Profiling Interactive Analysis) analysis. MTA2 was significantly positively correlated with both MMP2 expression (R = 0.37, P < 0.001; Figure 8A) and p38MAPK expression (R = 0.47, P < 0.001; Figure 8B). These results suggest that MTA2 contributes to the tumor metastasis of HCC cells by activating the p38MAPK/MMP2 pathways.

## Discussion

HCC remains a severe form of cancer with a low survival rate worldwide. Advanced approaches, including chemotherapy, surgical resection, radiotherapy, and liver transplantation, have thus far been unsuccessful in treating patients with HCC. It is clear that novel approaches must be urgently explored to treat HCC and to prevent the recurrence and metastasis of cancerous growth by identifying therapeutic targets for HCC. The findings of the present study indicated the following: (i) MTA2 expression is correlated with poor OS in patients with HCC. (ii) MTA2 knockdown by shMTA2 considerably reduced the cell invasion and mobility of human HCC cells; however, MTA2 knockdown exerted no influence on the induction of cell cycle arrest or the cell survival. (iii) MTA2 knockdown reduced the promoter activity, mRNA expression, and protein level of MMP-2 in HCC cells. (iv) The activation of the p38MAPK signaling pathway mediated MTA2-shRNA-downregulated migration and invasion in human HCC cells (Figure 8C). It was determined that MTA2 is an oncogene and a poor prognostic factor for the OS of HCC patients and that changes in p38MAPK-mediated MMP2 expression may contribute to tumor metastasis.

Studies have reported the upregulation of MTA2 in many types of cancers, such as gastric cancer 14, 15, breast cancer 11, and non-small-cell lung carcinoma (NSCLC) 16. MTA2 was observed to be highly expressed in pancreatic ductal adenocarcinoma (PDAC) and correlated with aggressive properties and poor prognosis 17. Similar reports have demonstrated the possible role of MTA2 in the progress of NSCLC 16. For molecular regulation, MTA2 acts as a critical factor in the Twist-mediated metastasis of mammary tumors 18. In the present study, it was MTA2 protein was overexpressed in HCC tissues and correlated with higher grade of HCC. MTA2 was significantly associated with poor OS in patients with HCC, and the findings suggested that MTA2 acts as an independent predictive factor for HCC.

ECM remodeling by MMPs contributes to tumor cell invasion and metastasis, finally leading to the development of a malignant tumor 19. A higher MMP2 level and MMP2/TIMP2 ratio in serum can indicate poor prognosis after transhepatic arterial chemoembolization, suggesting that MMPs are potential biomarkers for prognosis in HCC 20. The upregulation of MMP2 accelerates the progress of malignant tumors, indicates poor prognosis, and shortens the disease-free survival and OS in patients with HCC 19. Meanwhile, increases in mRNA as well as MMP2 and MMP9 serum levels have been found in patients with liver diseases and HCC 19, 21, 22. The serum MMP9/MMP2 ratio could be used as an accessory diagnostic marker in hepatitis-B-virus-related HCC. This ratio might also be useful in distinguishing between patients with early stage or advanced HCC 21. Some evidence demonstrated inhibition of human glioma cell migration and invasion abilities through a reduction in MMP2/MMP9 expression through MTA2 knockdown 13. Indeed, reduction in MMP2 by myeloid zinc finger 1 (MZF1) considerably suppresses migration and metastasis in human cervical cells 23. Another study revealed that the knockdown of endothelial cell specific molecule-1 promoted tumorigenicity and metastasis through the regulation of MMP2/MMP9 in prostate cancer cells 24. Therefore, we proposed that the knockdown of MTA2 downregulates the expression of MMP2, inhibiting tumor metastasis in HCC.

Delire and Starkel found that high MAPK pathway signaling in 50%-100% of human HCCs and determined that it correlated with poor prognosis 25. The p38 MAPK protein acts as a key factor in breast cancer, with p38 promoting breast cancer progression and lung metastasis by enhancing cell proliferation and detachment. The loss of p38 results in a reduction in primary tumor size and limits the potential for metastasis to the lungs 26. The p38 MAPK pathway was also shown to have a predominant role in colorectal cancer development and chemoresistance. Blockage of this pathway may have therapeutic potential in clinical settings 27. Moreover, the inhibition of dual MEK and p38 MAPK could be a potent therapeutic strategy against NSCLC driven by the KRAS oncogene 28. Capsaicin could induce apoptosis in KSHV-positive primary effusion lymphoma through the suppression of ERK and p38MAPK signaling and IL-6 expression 29. Moreover, p38MAPK is involved in protein kinase C alpha-regulated invasion in human HCC cells 30 and may be used as a diagnostic or prognostic marker of NSCLC 31. Downregulation of MTA2 through the suppression of both the PI3K/AKT and MAPK/ERK pathways ultimately leads to the inhibition of breast tumor growth 32. Silencing MTA2 inhibits the invasive potential of NSCLC cells through the ERK/AKT and VEGF signaling pathways 33. In the present study, the inhibitory effect of MTA2 knockdown on the migration and invasion of HCC cells by inactivating the p38MAPK signaling pathway was confirmed.

The present study suggests that MTA2 plays a critical role in HCC progression. MTA2 was expressed in higher amounts in human HCC tissues compared with adjacent normal tissues. High expression of MTA2 was also correlated with low OS in patients with HCC. MTA2 knockdown in HCC cells considerably inhibited the capacity for migration and invasion and reduced MMP2 expression. In addition, the activity of the p38 MAPK signaling pathway was found to mediate the MTA2- downregulated decrease in invasive motility in human HCC cells.

## Figures and Tables

**Table 1:**
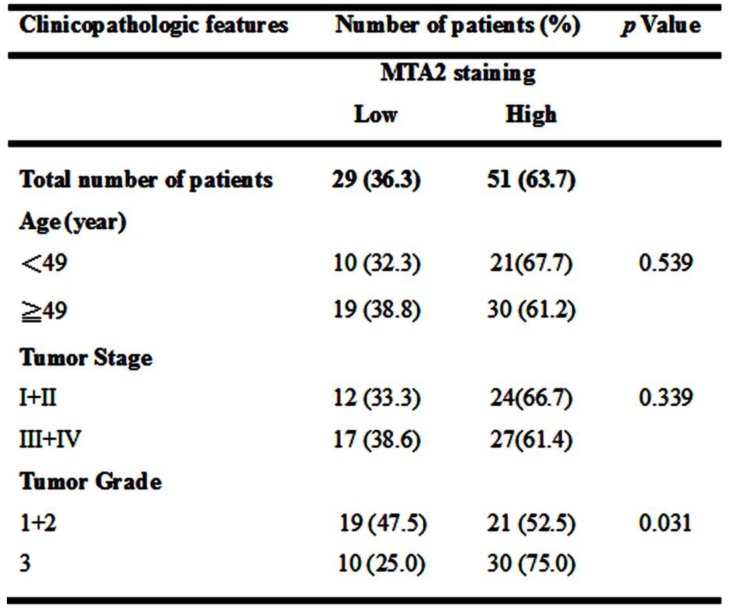
Relationship between MTA2 expression and clinicopathologic characteristics of HCC patients

## Abbreviations

HCC: Hepatocellular Carcinoma
MTA2: Metastasis tumor-associated protein 2
IHC: Immunohistochemistry
MAPK: mitogen-activated protein kinase
p38 MAPK: *p38* mitogen-activated *protein* kinases
MMP-2: Matrix Metallopeptidase-2
MMP-9: Matrix Metallopeptidase-9
EMT: *epithelial-mesenchymal transition*
MTT: 3-(4,5-Dimethylthiazol-2-yl)-2,5-diphenyl-tetrazolium bromide
shRNA: short hairpin RNA
siRNA: small interfering RNA
